# Biological invasions: a field synopsis, systematic review, and database of the literature

**DOI:** 10.1002/ece3.643

**Published:** 2013-06-12

**Authors:** Edward Lowry, Emily J Rollinson, Adam J Laybourn, Tracy E Scott, Matthew E Aiello-Lammens, Sarah M Gray, James Mickley, Jessica Gurevitch

Ecology and Evolution 3:181–196

DOI: 10.1002/ece3.431

On page 196, we missed adding the spreadsheets of Appendices 4, 5, and 6 to the online version of this article. We apologize for this and regret this oversight.

The full article, with the complete Appendices, can be viewed at http://onlinelibrary.wiley.com/doi/10.1002/ece3.431/full.

